# Supplementary figures and images for: Combination of serum CST1 and HE4 for early diagnosis of endometrial cancer
Source: PeerJ. 2023 Dec 5;11:e16424. doi: 10.7717/peerj.16424 (PMC10704982; doi:10.7717/peerj.16424)

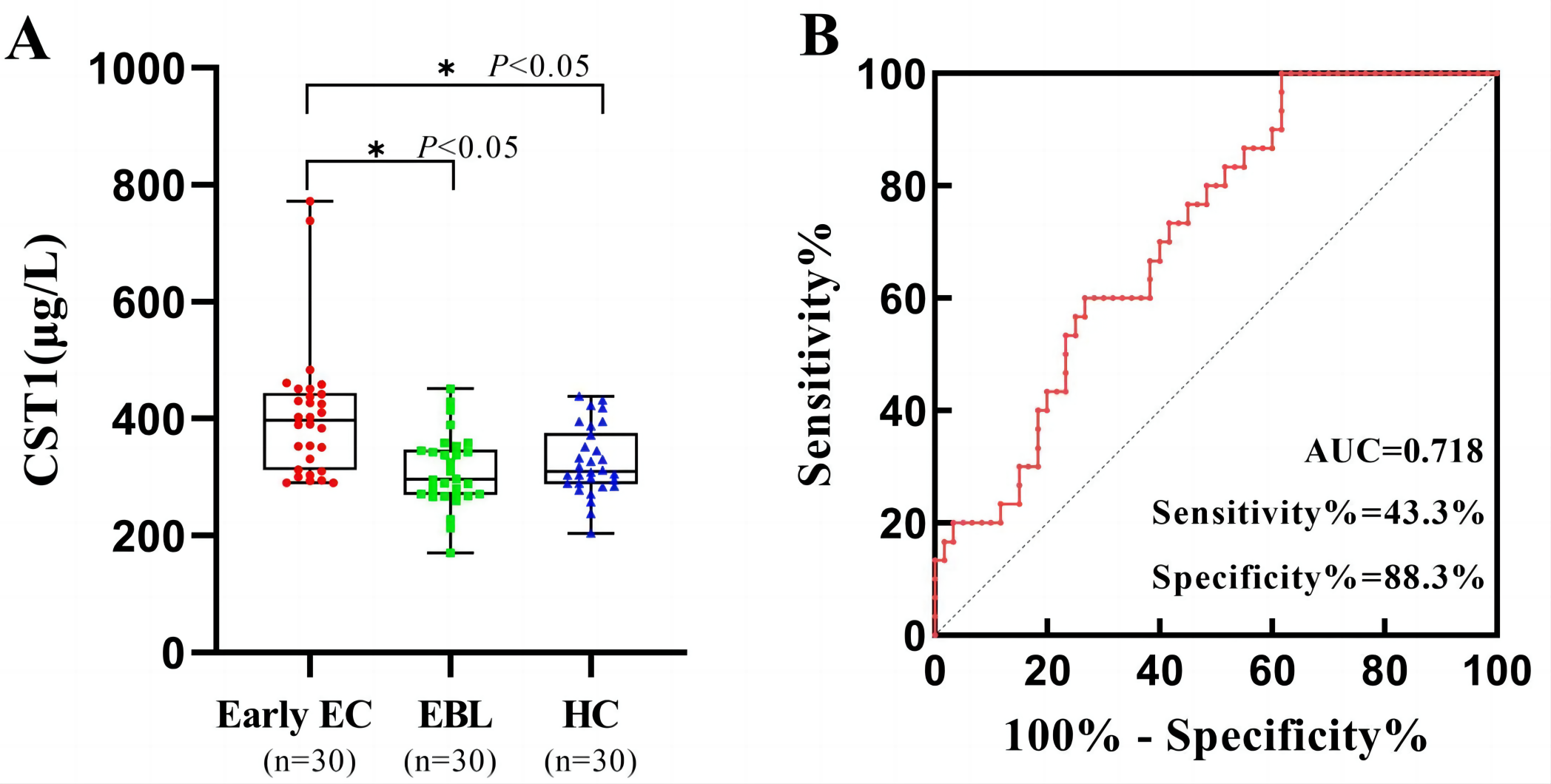

Supplement: Supplemental Information 1 — Boxplot, scatter of serum CST1 (A), and the ROC curve of serum CST1(B). [file peerj-11-16424-s001.png]
