# Supplementary material for: Combination of serum CST1 and HE4 for early diagnosis of endometrial cancer
Source: PeerJ. 2023 Dec 5;11:e16424. doi: 10.7717/peerj.16424 (PMC10704982; doi:10.7717/peerj.16424)
Supplement: Supplemental Information 2 [file peerj-11-16424-s002.docx]

**Supplemental Table 1**

Results of detection limit of the CST1 kit

| No. | CST1 (μg/L) | No. | CST1 (μg/L) |
| --- | --- | --- | --- |
| 1 | 3.208 | 11 | 3.153 |
| 2 | 3.029 | 12 | 3.104 |
| 3 | 3.012 | 13 | 2.975 |
| 4 | 3.119 | 14 | 2.997 |
| 5 | 3.114 | 15 | 2.964 |
| 6 | 3.060 | 16 | 2.980 |
| 7 | 3.027 | 17 | 3.176 |
| 8 | 3.065 | 18 | 3.010 |
| 9 | 3.427 | 19 | 3.028 |
| 10 | 3.159 | 20 | 3.039 |
| Mean value () 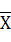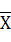 | 3.082 | | |
| Standard deviation | 0.106 | | |
| （+2SD） 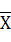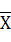 | 3.294 | | |

SD: Standard deviation.

**Supplemental Table 2**

Linearity test results of the CST1 kit

| Concentration of CST1 calibrator（μg/L） | 0 | 50 | 100 | 200 | 400 | 800 | 1600 |
| --- | --- | --- | --- | --- | --- | --- | --- |
| Tested optical density | 0.051 | 0.178 | 0.217 | 0.301 | 0.485 | 0.852 | 1.569 |
|  | 0.053 | 0.172 | 0.223 | 0.305 | 0.491 | 0.842 | 1.557 |
| Mean optical density | 0.052 | 0.175 | 0.220 | 0.309 | 0.488 | 0.847 | 1.563 |

**Supplemental Table 3**

Results of recovery test of the CST1 kit

|  | Mixed sample 1 | | Mixed sample 2 | | Mixed sample 3 | | Diluted serum sample | | |
| --- | --- | --- | --- | --- | --- | --- | --- | --- | --- |
|  | 1 | 2 | 1 | 2 | 1 | 2 | 1 | 2 | 3 |
| Tested results (μg/L) | 67.58 | 68.20 | 135.31 | 136.73 | 228.17 | 230.05 | 63.48 | 64.07 | 64.36 |
| Mean results (μg/L) | 67.89 | | 136.02 | | 229.11 | | 63.97 | | |
| Recovery rate (%) | 103.19 | | 98.06 | | 107.21 | |  |  |  |
| Mean(%) | 102.82 | | | | | |  |  |  |

**Supplemental Table 4**

Intra-batch precision test results of the CST1 kit

| Tumor marker | CST1 (μg/L) | |
| --- | --- | --- |
| Times | Results of low value | Results of high value |
| 1 | 267.44 | 396.24 |
| 2 | 243.56 | 397.57 |
| 3 | 230.08 | 412.44 |
| 4 | 247.14 | 420.29 |
| 5 | 241.35 | 429.57 |
| 6 | 246.53 | 427.12 |
| 7 | 256.36 | 448.01 |
| 8 | 265.19 | 459.81 |
| 9 | 237.49 | 396.37 |
| 10 | 234.98 | 396.06 |
| 11 | 225.19 | 421.43 |
| 12 | 246.5 | 416.65 |
| 13 | 230.98 | 402.95 |
| 14 | 231.92 | 414.21 |
| 15 | 247.97 | 420.53 |
| 16 | 230.52 | 449.76 |
| 17 | 247.71 | 333.32 |
| 18 | 245.64 | 331.73 |
| 19 | 230.67 | 354.46 |
| 20 | 245.28 | 357.46 |
| Mean value (U/ml) | 242.63 | 404.29 |
| Standard deviation (U/ml) | 11.23 | 35.05 |
| Coefficient of variation(%) | 4.63 | 8.67 |

**Supplemental Table 5**

Inter-batch precision test results of the CST1 kit

| Tumor marker | CST1 (μg/L) | | |
| --- | --- | --- | --- |
| Days | Times | Results of low value | Results of high value |
| 1 | 1 | 228.32 | 310.35 |
|  | 2 | 202.75 | 323.71 |
|  | 3 | 223.73 | 339.46 |
| 2 | 4 | 216.41 | 271.18 |
|  | 5 | 244.29 | 303.15 |
|  | 6 | 200.19 | 280.71 |
| 3 | 7 | 219.63 | 284.47 |
|  | 8 | 212.59 | 279.03 |
|  | 9 | 198.64 | 273.23 |
| 4 | 10 | 218.69 | 265.62 |
|  | 11 | 216.16 | 305.06 |
|  | 12 | 216.47 | 287.95 |
| 5 | 13 | 197.41 | 302.11 |
|  | 14 | 212.48 | 329.73 |
|  | 15 | 236.87 | 350.97 |
| Mean value (U/ml) | | 216.31 | 301.12 |
| Standard deviation (U/ml) | | 13.07 | 22.75 |
| Coefficient of variation (%) | | 6.04 | 7.56 |

**Supplemental Table 6**

Results of interference test of the CST1 kit

|  | No.1 | | | No.2 | | |
| --- | --- | --- | --- | --- | --- | --- |
|  | Results  (μg/L) | The control  (μg/L) | Bias value  (%) | Results  (μg/L) | The control  (μg/L) | Bias value  (%) |
| Hemoglobin | 50.94 | 48.50 | 5.03 | 94.01 | 87.61 | 7.31 |
| Bilirubin | 52.25 | 48.50 | 7.73 | 93.26 | 87.61 | 6.45 |
| Triglyceride | 53.19 | 48.50 | 9.68 | 95.66 | 87.61 | 9.19 |
